# Influence of Calcium-Sequestering Salts on Heat-Induced Changes in Blends of Skimmed Buffalo and Bovine Milk

**DOI:** 10.3390/foods12112260

**Published:** 2023-06-03

**Authors:** Carolyn T. Mejares, Jayani Chandrapala, Thom Huppertz

**Affiliations:** 1 School of Science, STEM College, RMIT University, Melbourne, VIC 3083, Australia; carolyn.mejares@rmit.edu.au (C.T.M.); jayani.chandrapala@rmit.edu.au (J.C.); 2 School of Technology, University of the Philippines Visayas, Miagao 5023, Iloilo, Philippines; 3 FrieslandCampina, 3818 LE Amersfoort, The Netherlands; 4 Food Quality and Design Group, Wageningen University & Research, 6708 WG Wageningen, The Netherlands

**Keywords:** buffalo milk, milk mixture, calcium-sequestering salt, heat treatment, protein

## Abstract

Heat-induced interactions of calcium and protein in milk lead to undesirable changes in the milk, such as protein coagulation, which can be minimized through the addition of calcium-sequestering salts prior to heat treatment. Thus, the present study investigated the influence of 5 mM added trisodium citrate (TSC) or disodium hydrogen phosphate (DSHP) on the heat-induced (85 °C and 95 °C for 5 min) changes in physical, chemical, and structural properties of buffalo and bovine skim milk mixtures (0:100, 25:75, 50:50, 75:25, and 100:0). Significant changes in pH and calcium activity as a result of TSC or DSHP addition subsequently resulted in higher particle size and viscosity as well as non-sedimentable protein level. These changes are mostly observed during heat treatment at 95 °C and increased proportionally to the concentration of buffalo skim milk in the milk mixture. Significant changes were affected by TSC addition in the 75:25 buffalo:bovine milk blend and buffalo skim milk, but for other milk samples, TSC addition effected comparable changes with DSHP addition. Overall, the addition of TSC or DSHP before heat treatment of buffalo:bovine milk blends caused changes in milk properties that could reduce susceptibility of milk to coagulation.

## 1. Introduction

Milk is one of the oldest natural beverages and is considered a complete food [1]. There is considerable interest in buffalo milk due to its high nutritional value compared to bovine milk. For instance, buffalo milk generally has a higher protein content (4.35%) than bovine milk (3.35%), and the total calcium concentration has also been found to be higher in buffalo milk (47.1 mM) than in bovine milk (30.5 mM) [2]. Calcium in milk is distributed between the colloidal and soluble phases of milk. In bovine milk, ~65% of total calcium is associated with casein micelles [3], whereas ~80% of total calcium is in the micellar phase in buffalo milk [2]. The interaction of calcium ions with milk proteins can lead to changes in the physicochemical properties of milk, such as coagulation of proteins during thermal treatment [4]. One approach to minimize undesirable effects from calcium–protein interactions during thermal processing of milk is through the addition of calcium-sequestering salts (CSSs) to milk prior to processing [5].

A wide range of CSSs, such as orthophosphates, citrates, and hexametaphosphates, can be used to improve the heat stability or to improve the technological properties of dairy products [5,6,7]. The addition of calcium sequestrants to milk results in an alteration of the mineral balance, e.g., leading to a decrease in the concentration of free calcium ions, depletion of colloidal calcium phosphate from the micelles, and release of specific caseins from the micelle [8,9,10,11,12]. These changes can induce various modifications to casein micelles and consequently on milk characteristics [9,10]. For instance, the depletion of colloidal calcium phosphate from micelles leads to the dissociation and release of casein fractions into the aqueous phase, where it binds with calcium ions, thereby improving the heat stability of milk products [13].

Heat-treated buffalo milk and dairy products from buffalo and bovine milk mixtures are commonly marketed in Asian countries. However, there are very few studies on the addition of CSSs and their effects on buffalo milk or their mixtures with bovine milk. Solanki and Gupta [14] reported an increase in heat stability of buffalo skim milk ultrafiltration retentate (BSMUR) after the addition of 0.20% monosodium phosphate. Sindhu [15] also reported an increased heat stability of concentrated buffalo milk after the addition of up to 0.1% monosodium phosphate on the fluid milk before heat treatment and concentration. Additionally, the heat stability of a dairy whitener produced from BSMUR was enhanced by the addition of 0.4% of a 2:1 (*w*/*w*) mixture of monosodium and disodium phosphate [16]. As protein and calcium concentrations of buffalo milk differ from those in bovine milk, it is necessary to explore how CSSs affect the properties of buffalo skim milk and mixtures with bovine skim milk. This would further support realization of the production potential of buffalo milk. Hence, this study aimed to determine changes in the physicochemical and structural properties of buffalo skim milk and mixtures with bovine skim milk (0:100, 25:75, 50:50, 75:25, and 100:0 ratios) with the addition of trisodium citrate (TSC; 5 mM) or disodium hydrogen phosphate (DSHP; 5 mM) prior to heat treatments at 85 °C and 95 °C for 5 min. These heating conditions were based on our previous study [17] where significant changes in milk physicochemical properties were observed at heat treatments ≥ 85 °C.

## 2. Materials and Methods

### 2.1. Materials

Raw buffalo and bovine milk were provided by Floridia Cheese (Thomastown, VIC, Australia) and Saputo Dairy Australia (Laverton North, VIC, Australia), respectively. Raw milk was added with 0.02% sodium azide (CAS No. 26628-22-8; Sigma-Aldrich, St. Louis, MO, USA) and stored under refrigerated conditions until analysis the next day. Trisodium citrate dihydrate (CAS No. 6132-04-3; Na_3_C_6_H_5_O_7_∙2H_2_O) and disodium hydrogen phosphate dodecahydrate (CAS No. 10039-32-4; Na_2_HPO_4_∙12H_2_O) were purchased from Sigma-Aldrich (Australia).

### 2.2. Sample Preparation

Skimming of milk was as previously described [17]. Milk blend samples were then prepared by mixing buffalo skim milk and bovine skim milk in desired proportions (0:100, 25:75, 50:50, 75:25, and 100:0, *v*/*v*). Each of the milk samples were further divided into three lots, where the first lot served as the control with no addition of CSSs, the second lot with the addition of 5 mM TSC, and the third lot with the addition of 5 mM DSHP. At this CSS concentration, pronounced increases in the heat coagulation time were previously reported [18]. The solutions were mixed for 15 min prior to heat treatment.

### 2.3. Thermal Treatment

Milk samples in 100 mL aliquots were transferred to aluminium containers and heated at 85 °C and 95 °C for 5 min in a thermostatically controlled water bath. A come-up time of 6 min and 10 min, respectively, was recorded. Heat-treated milk samples were immediately immersed in an ice bath and transferred to plastic containers upon reaching a temperature of 10 °C then stored under refrigeration. The milk samples were allowed to equilibrate to 20 °C prior to analyses.

### 2.4. Analytical Measurements

#### 2.4.1. Particle Size and Zeta Potential Measurements

Particle size and ζ-potential of samples diluted fourfold with Milli-Q water were analysed at 20 °C using Malvern Zetasizer Nano ZS instrument (ZEN3600, Malvern Instruments Ltd., Malvern, UK). Five readings from each individual sample were collected.

#### 2.4.2. Measurement of pH, Calcium Activity, and Viscosity

A digital pH meter (SevenCompact pH/Ion, Mettler Toledo, Columbus, OH, USA) equipped with an automatic temperature compensation InLab^®^ Max Pro-ISM probe, which was calibrated at 20 °C using buffers at pH 4, 7, and 10, was used to measure the pH of milk samples. The calcium ion activity was measured at 20 °C using a perfectION™ combined calcium ion selective electrode, while viscosity was determined using a Discovery Hybrid rheometer (HR-2, TA Instruments, New Castle, DE, USA) operated by TRIOS software as described previously [17]. Measurements were made in triplicate.

### 2.5. Protein Distribution

Milk samples were initially centrifuged at 88,000× *g* for 60 min at 20 °C in a Beckman ultracentrifuge and the associated Kontron TFT 50.13 fixed-angle rotor. A clear supernatant which contains non-sedimentable proteins was carefully removed from the pellet and analysed by reduced SDS-PAGE following a procedure described previously [17]. Protein bands were visualized using ChemiDoc MP Imaging System (Bio-Rad, Hercules, CA, USA) and the intensities of major protein bands were determined using ImageJ software (version 8) to calculate the quantity of individual proteins.

### 2.6. Data and Statistical Analysis

The experiment was carried out in two separate milk batches. To assess whether sequestering salt and heat treatment significantly affect skim milk properties, analytical data were subjected to two-way analysis of variance (ANOVA) using SPSS statistical software version 28.0 (IBM, New York, NY, USA). Mean differences were analysed using Tukey’s probability pairwise comparison test at *p* < 0.05.

## 3. Results

### 3.1. Physicochemical Changes of Heat-Treated Milk with the Pre-Heat Addition of CSSs

#### 3.1.1. Particle Size and ζ-Potential Changes

The particle size of bovine skim milk was significantly lower than buffalo skim milk, and blending buffalo and bovine skim milk changed particle size proportionally to the addition of buffalo skim milk irrespective of heat treatment or CSS addition (Table 1). In unheated samples, the particle size significantly increased with the addition of both CSSs for all ratios except for the 50:50 buffalo:bovine milk blend, where only TSC caused a significant increase in particle size. The magnitude of the increases in size with the addition of TSC or DSHP were similar for most ratios except for buffalo milk where the increase in size was significantly higher with the addition of DSHP compared to the addition of TSC. Similarly, DSHP addition caused a significant change (less negative) in the zeta potential of unheated 25:75 and 50:50 buffalo:bovine milk mixtures as well as bovine skim milk (Table 1).

Heat treatment of control milk did not induce significant size and zeta potential changes in milk samples except bovine skim milk, where the zeta potential significantly increased (more negative) with heating at 85 °C and 95 °C. Similar to the control (no CSS addition), an increasing trend in zeta potential (more negative) was observed with heating CSS-added milk. This is in contrast to heat-induced changes in particle size, where a decreasing trend was observed in all DSHP-added milk samples and TSC-added bovine skim milk, 25:75, and 50:50 buffalo:bovine milk blends heated at 85 °C and 95 °C. Although TSC addition resulted in an increased particle size of buffalo skim milk and the 75:25 buffalo:bovine milk blend during heat treatment, such changes were not significant. Whilst CSS addition and heat treatment caused significant reduction in the particle size of milk samples, the values were still significantly higher than heated control samples. This is particularly evident from the TSC-added buffalo skim milk, 25:75, and 75:25 buffalo:bovine milk blends heated at 95 °C. In terms of zeta potential, significant heat-induced increases (more negative) were only measured in CSS-added bovine skim milk, 25:75, and 50:50 buffalo:bovine milk blends as well as DSHP-added 75:25 milk blend with heating at 85 °C and 95 °C. Nevertheless, zeta potential values of heat-treated control and CSS-added milk samples were comparable (Table 1).

#### 3.1.2. Changes in pH and Ca^2+^ Activity

Addition of either TSC or DSHP significantly increased the pH and decreased the Ca^2+^ activity of unheated samples irrespective of the milk ratio (Table 2). Similar to the control samples (no CSS addition), a decreasing trend in pH and Ca^2+^ activity was observed in CSS-added milk samples during heat treatment. Notably, all samples showed a significant drop in pH after heating at 95 °C, regardless of the salt added. Likewise, heat treatment at 85 °C caused a significant pH reduction in TSC-added bovine skim milk and 25:75 buffalo:bovine milk blend. Regardless of differences in the extent of pH reduction with heating, TSC-added milk had significantly higher pH values than the control and DSHP-added counterpart (Table 2).

In terms of Ca^2+^ activity, a significant reduction was measured in all DSHP-added milk with heating at 85 °C and 95 °C. TSC addition on the other hand only effected significant Ca^2+^ activity reduction with heating at 95 °C for pure milk and 25:75 buffalo:bovine milk blend (Table 2). However, a significantly higher reduction in Ca^2+^ activity was effected by DSHP addition and heat treatment at 85 °C than TSC did in 25:75 and 75:25 buffalo:bovine milk blends but comparable values in other milk samples. Similarly, DSHP addition in buffalo skim milk and 75:25 buffalo:bovine milk blend resulted in a significantly higher Ca^2+^ activity reduction with heating at 95 °C.

#### 3.1.3. Viscosity Changes

Addition of DSHP did not result in a significant increase in the viscosity of unheated milk, but TSC addition significantly increased the viscosity where the magnitude of increase was greater in pure milk than milk mixtures (Table 2). Heat treatment caused an increasing trend in viscosity of both CSS-added milk and the control samples. Regardless of the heating temperature applied, it appears that TSC influenced significant heat-induced viscosity increases in milk with a lower proportion of buffalo skim milk, while DSHP afforded such an effect in milk with higher proportion of buffalo skim milk. As shown in Table 2, a significant increase in viscosity was measured in TSC-added bovine skim milk and 25:75 buffalo:bovine milk blend with heating at 85 °C and 95 °C. At the same heating temperatures, DSHP-added buffalo skim milk, 50:50, and 75:25 buffalo:bovine milk blend also exhibited significant viscosity increases. Additionally, the DSHP-added 25:75 buffalo:bovine milk blend also showed a significant increase in viscosity after heating at 95 °C. Regardless of these changes, the viscosity of TSC-added milk was comparable to DSHP-added milk upon heating except in buffalo skim milk, where TSC addition effected a significantly higher viscosity upon heating at 95 °C.

### 3.2. Influence of CSSs on Heat-Induced Changes in Protein Distribution

**Figure 1:**
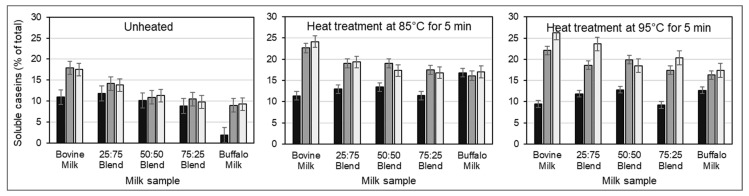
Changes in the level of non-sedimentable caseins in skim milk without the addition of CSSs (∎) or with the addition of TSC (∎) and DSHP (□) and subsequent heating. Milk blends refer to the ratio of buffalo skim milk to bovine skim milk. Error bars represent standard deviations.

**Figure 2:**
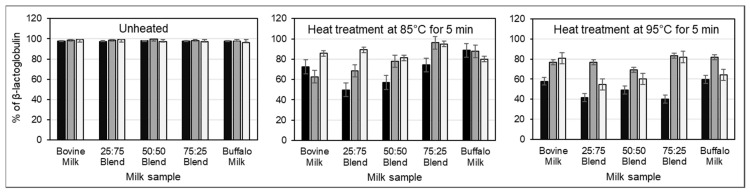
Changes in the level of non-sedimentable β-LG in skim milk without the addition of CSSs (∎) or with the addition of TSC (∎) and DSHP (□) and subsequent heating. Milk blends refer to the ratio of buffalo skim milk to bovine skim milk. Error bars represent standard deviations.

**Figure 3:**
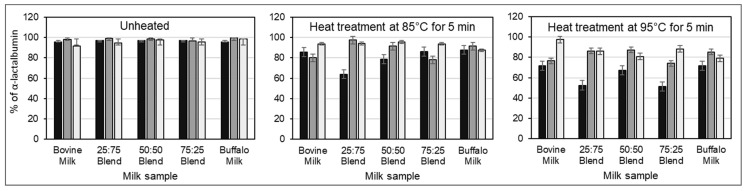
Changes in the level of non-sedimentable α-LA in skim milk without the addition of CSSs (∎) or with the addition of TSC (∎) and DSHP (□) and subsequent heating. Milk blends refer to the ratio of buffalo skim milk to bovine skim milk. Error bars represent standard deviations.

The addition of CSSs before heating increased the level of non-sedimentable caseins in both unheated and heated milk (Figure 1). While heating alone induced maximum casein dissociation at 85 °C, addition of CSSs before heat treatment resulted in a continuous increase in the level of non-sedimentable caseins as heating temperature increased from 85 °C to 95 °C. The heat-induced reduction in the level of non-sedimentable whey proteins was greatly minimized by the addition of CSSs prior to heating, but this was dependent on the buffalo:bovine milk ratio, heating temperature, and the type of CSS (Figure 2 and Figure 3). Notably, the 25:75 and 50:50 buffalo:bovine milk blends showed the lowest level of non-sedimentable β-lactoglobulin (β-LG) after heat treatment at 95 °C, irrespective of the CSS added (Figure 2). Buffalo skim milk and 75:25 buffalo:bovine milk blend had the highest level of non-sedimentable β-lactoglobulin (β-LG) with the addition of TSC and heating at 85 °C and 95 °C. A reduction in the level of non-sedimentable α-lactalbumin (α-LA) on the other hand was lower with addition of DSHP in bovine skim milk and milk blends irrespective of heating temperature (Figure 3). In contrast, less reduction in the level of non-sedimentable α-LA was afforded by TSC in buffalo skim milk.

## 4. Discussion

Interactions of sequestrants with calcium ions in milk result in a shift in protein–mineral equilibrium, which subsequently led to a significant reduction in Ca^2+^ activity concurrent to a pH increase in unheated milk (Table 2) in accord with the results of other authors [19,20]. Milk pH progressively increased and Ca^2+^ activity decreased with increasing proportion of buffalo skim milk in the milk mixture, but the extent of CSS-induced pH or Ca^2+^ activity changes was greater as the concentration of bovine skim milk increased (Table 2). Bovine milk has a lower buffering capacity and a higher percentage of soluble calcium (~35%) than buffalo milk (~20% soluble calcium) [2,3], which mostly would explain the observed pH and Ca^2+^ activity differences in these milks. Irrespective of milk ratio, the magnitude of pH increase was significantly higher with the addition of TSC compared to DSHP (Table 2). While both CSSs sequester calcium, TSC dissolves colloidal calcium and remains as stable, soluble complexes in the serum phase, whereas complex of DSHP with calcium precipitates in the casein micelle [21,22]. Therefore, to re-establish equilibrium between HPO_4_^2−^ and H_2_PO_4_^−^/PO_4_^3−^ in the serum phase, some H^+^ ions are released, thus resulting in significantly lower pH in DSHP-added milk. Although a significant difference in pH change was exhibited by these calcium sequestrants, the decrease in Ca^2+^ activity of unheated milk was comparable between TSC and DSHP, suggesting that TSC and DSHP have similar calcium-binding ability, in agreement with previous reports [20,23]. The decrease in Ca^2+^ activity with increasing proportion of buffalo skim milk in the unheated milk samples was strongly correlated (R^2^ = −0.92 to −0.99) with a progressive increase in milk pH irrespective of salt addition.

With heating, a significant reduction in pH and Ca^2+^ activity was observed at 95 °C irrespective of milk ratio and type of salt added. However, the extent of pH and Ca^2+^ activity reductions during heating of milk blends at 95 °C increased as the concentration of buffalo skim milk increases in control and DSHP-added milk but was similar for TSC-added milk (Table 2). Calcium and phosphate in milk are distributed between the aqueous and micellar phases, wherein ~80% and ~65% of total calcium and 66% and 48% of inorganic phosphate were in the micellar phases of buffalo and cow milk, respectively [2,3]. Heat treatment affects this equilibrium whereby soluble calcium and phosphate is converted into the micellar phase [24,25], leading to saturation of the colloidal calcium phosphate and thus precipitation as either monocalcium phosphate or tricalcium phosphate [26,27]. As buffalo milk contains a higher concentration of micellar calcium phosphate, re-establishment of equilibrium would require more HPO_4_^2-^ and PO_4_^3-^ ions to be released, which would explain greater pH reduction in milk blends having a higher proportion of buffalo skim milk.

The shift in protein–mineral equilibrium due to calcium sequestration results in dissolution of colloidal calcium phosphate and release of specific caseins from the micelles [8,11] or dissociation of casein micelles into small clusters [10,12]. This is evident in SDS-PAGE data where unheated samples with added CSS have higher levels of non-sedimentable caseins than the control samples without added CSS (Figure 1). Although the level of non-sedimentable caseins decreased with increasing proportion of buffalo skim milk, the increases in particle size and viscosity (Table 2) were higher in unheated milk blends containing higher concentration of buffalo skim milk irrespective of the type of salt added. Caseins in buffalo milk are mostly in micellar form, more mineralized, and are larger in size than bovine milk [1,28,29]. On addition of CSSs, the more mineralized casein micelles of buffalo milk would be mostly affected, hence greater casein dissociation. Some of these dissociated casein micelles however were assumed to be large enough to co-sediment with intact micelles during ultracentrifugation, hence the level remaining in the serum phase were lower than that of bovine milk (Figure 1). The significant increases in size and viscosity of unheated TSC-added buffalo milk and milk blends despite having lower levels of non-sedimentable caseins than bovine milk could probably be related to swelling of the casein micelles. Calcium–CSS complex formation prevents the interaction of calcium with caseins, hence inhibiting aggregation [20]. Citrate solubilizes colloidal calcium phosphate, leading to a reduction in the level of bound phosphate, decreased interactions between caseins, and thus results in more hydrated protein matrix [11,30]. It would seem that swelling, rather than dissociation of smaller casein clusters, contributes largely to significant viscosity increase, because DSHP and TSC addition resulted in a comparable size increase (Table 1), but only TSC addition effected a significant viscosity increase (Table 2).

Heat treatment also induces alteration of salt equilibrium in milk leading to casein dissociation and diffusion into the serum phase [23,31]. Additionally, whey proteins are denatured during heating, which could form aggregates with themselves or with caseins. Attachment of the whey protein, particularly β-LG, to the casein micelle surface would result in a more negative zeta potential value [32], which would explain the observed zeta potential changes in CSS-added 25:75 and 50:50 buffalo:bovine milk blends as well as bovine skim milk (Table 1). Heat-induced significant changes in zeta potential were not observed in milk having higher proportion of buffalo skim milk probably because whey proteins in buffalo milk are more heat-stable than bovine milk whey proteins. On mixing these milks, casein–whey protein aggregation would occur faster due to the presence of heat-labile bovine milk whey proteins and higher casein surface for adsorption afforded by buffalo milk caseins [17]. This is further supported by the low level of non-sedimentable β-LG in 25:75 and 50:50 buffalo:bovine milk blend as shown in the SDS-PAGE data (Figure 2).

In addition to zeta potential changes, the heat-induced aggregation of denatured whey proteins to casein micelles, along with CSS-induced casein dissociation, also contributed to the observed size and viscosity changes of milk blends subjected to heat treatment. The significant reduction in particle size of DSHP-added milk blends with heating (Table 2) would mainly be attributed to dissociated smaller casein clusters and to a lesser extent at the formation of casein–whey protein complex. This is supported by a highly negative correlation of particle size to the level of non-sedimentable caseins (R^2^ = −94 to −0.99) but slightly low correlation with the level of non-sedimentable β-LG (R^2^ = 0.82–0.92). Although particle size decreased with heating, viscosity significantly increased proportionally to the concentration of buffalo skim milk in the milk mixture. It appears though that in milk blends containing a lower proportion of buffalo skim milk, the heat-induced viscosity increase was primarily due to swelling of the casein micelles, while aggregation of denatured whey proteins with casein had a small but measurable effect in viscosity. This is based on the fact that significant viscosity increases were measured in TSC-added bovine milk and 25:75 buffalo:bovine milk blends and in DSHP-added 50:50, 75:25 buffalo:bovine milk blends, and buffalo skim milk systems. Furthermore, SDS-PAGE data (Figure 1) showed that the increase in non-sedimentable caseins was higher in CSS-added buffalo milk and milk blends (7–11% increase) than CSS-added bovine milk (5–9% increase) which mostly would explain the larger size and higher viscosities observed with heating for these ratios.

## 5. Conclusions

The effect of TSC or DSHP addition prior to heating on physicochemical and structural changes of buffalo skim milk and mixtures with bovine skim milk can differ considerably due to inherent compositional variation in milk as well as differences in the formed complexes of these calcium sequestrants. Significant changes in pH and Ca^2+^ activity due to the alteration in protein–mineral equilibrium as a result of CSS addition and heat treatment mostly influenced heat-induced changes in size, viscosity, and protein denaturation and aggregation that occur during heating at 95 °C. TSC addition effected significant heat-induced changes on the physicochemical and structural properties of milk samples with a higher proportion of buffalo skim milk, but changes were comparable to DSHP addition in milk with lower proportion of buffalo skim milk. Overall, this study has elucidated the feasibility of adding either TSC or DSHP to buffalo:bovine milk mixture with better properties contributing to reduced protein denaturation and aggregation if heat treatment is carefully controlled based on the milk ratio.

## Figures and Tables

**Table 1:** Particle size and ζ-potential of buffalo skim milk and buffalo:bovine milk blends with the addition of CSSs prior to heat treatment.

Milk Blend	CSSs Added	Heat Treatment			Heat Treatment		
		None	85 °C	95 °C	None	85 °C	95 °C
		Particle Size (nm)			Zeta Potential (mV)		
0:100	None	187.3 ± 3.5 ^Aa^	186.9 ± 2.9^Aa^	189.9 ± 5.0 ^Aa^	−19.4 ± 2.1 ^Aa^	−21.5 ± 1.3 ^Ba^	−21.7 ± 1.6 ^Ba^
	TSC	194.0 ± 7.4 ^Ab^	184.3 ± 5.6 ^Ba^	184.1 ± 5.3 ^Bb^	−17.0 ± 2.6 ^Ab^	−22.7 ± 1.5 ^Ba^	−23.3 ± 1.6 ^Ba^
	DSHP	193.9 ± 6.2 ^Ab^	182.8 ± 4.4 ^Ba^	184.2 ± 3.6 ^Bb^	−16.6 ± 3.2 ^Ab^	−22.1 ± 1.5 ^Ba^	−22.5 ± 1.4 ^Ba^
25:75	None	193.8 ± 6.4 ^Aa^	195.2 ± 2.7 ^Aa^	195.9 ± 5.6 ^Aa^	−20.3 ± 1.9 ^Aa^	−21.7 ± 1.3 ^Aa^	−22.0 ± 1.5 ^Aa^
	TSC	200.6 ± 7.9 ^Ab^	192.1 ± 4.4 ^Ba^	201.5 ± 3.7 ^Ab^	−19.9 ± 2.0 ^Aab^	−22.3 ± 1.6 ^Ba^	−23.1 ± 2.2 ^Ba^
	DSHP	201.8 ± 6.5 ^Ab^	195.1 ± 7.9 ^Ba^	195.4 ± 7.5 ^Ba^	−18.6 ± 2.5 ^Ab^	−22.1 ± 1.5 ^Ba^	−22.7 ± 1.8 ^Ba^
50:50	None	199.3 ± 6.3 ^Aa^	197.7 ± 4.1 ^Aa^	202.7 ± 3.4 ^Aa^	−20.6 ± 1.5 ^Aa^	−21.8 ± 1.5 ^Aa^	−22.3 ± 1.5 ^Aa^
	TSC	205.6 ± 9.2 ^Ab^	199.0 ± 6.8 ^Ba^	203.8 ± 3.7 ^ABa^	−20.5 ± 2.3 ^Aab^	−23.0 ± 1.6 ^Ba^	−22.2 ± 1.7 ^Ba^
	DSHP	202.8 ± 4.9 ^Aab^	200.2 ± 7.6 ^Aa^	199.8 ± 5.6 ^Aa^	−18.9 ± 2.9 ^Ab^	−22.7 ± 1.7 ^Ba^	−22.2 ± 2.4 ^Ba^
75:25	None	201.1 ± 4.8 ^Aa^	201.7 ± 3.9 ^Aa^	203.6 ± 4.1 ^Aa^	−21.1 ± 1.3 ^Aa^	−21.9 ± 1.5 ^Aa^	−22.2 ± 1.7 ^Aa^
	TSC	206.9 ± 9.9 ^Ab^	207.6 ± 2.8 ^Ab^	210.6 ± 4.6 ^Ab^	−21.3 ± 2.0 ^Aa^	−22.5 ± 1.9 ^Aa^	−22.5 ± 1.5 ^Aa^
	DSHP	209.8 ± 4.7 ^Ab^	202.6 ± 4.1 ^Bab^	205.2 ± 5.8 ^ABa^	−19.7 ± 2.5 ^Aa^	−22.9 ± 1.8 ^Ba^	−21.9 ± 1.7 ^Ba^
100:0	None	202.2 ± 4.2 ^Aa^	205.1 ± 3.9 ^Aa^	204.9 ± 4.5 ^Aa^	−21.3 ± 1.4 ^Aa^	−22.0 ± 1.1 ^Aa^	−22.3 ± 1.6 ^Aa^
	TSC	208.8 ± 8.0 ^Ab^	209.1 ± 3.1 ^Aa^	211.7 ± 3.6 ^Ab^	−21.7 ± 1.8 ^Aa^	−22.6 ± 2.4 ^Aa^	−22.4 ± 1.8 ^Aa^
	DSHP	218.6 ± 10.0 ^Ac^	206.8 ± 9.2 ^Ba^	208.0 ± 5.6 ^Bab^	−21.5 ± 1.6 ^Aa^	−22.5 ± 1.9 ^Aa^	−22.6 ± 1.8 ^Aa^

Values are means ± standard deviation. Within the same physical property, mean of samples in a row with different superscripts (A, B) differ significantly. Different superscripts (a, b, c) in the same column within the same type of milk indicate significant difference (*p* < 0.05). Milk blend refers to the ratio of buffalo skim to bovine skim milk.

**Table 2:** pH, Ca^2+^ activity, and viscosity of buffalo skim milk and buffalo:bovine milk blends following pre-heat addition of CSSs.

Milk Blend	CSSs Added	Heat Treatment			Heat Treatment			Heat Treatment		
		Unheated	85 °C	95 °C	Unheated	85 °C	95 °C	Unheated	85 °C	95 °C
		pH			Calcium Activity (mM)			Viscosity (mPa∙s)		
0:100	None	6.83 ± 0.02 ^Aa^	6.81 ± 0.02 ^ABa^	6.79 ± 0.02 ^Ba^	1.02 ± 0.07 ^Aa^	0.95 ± 0.05 ^ABa^	0.91 ± 0.06 ^Ba^	1.48 ± 0.20 ^Aa^	1.60 ± 0.15 ^ABa^	1.78 ± 0.09 ^Ba^
	TSC	7.02 ± 0.04 ^Ab^	6.97 ± 0.03 ^Bb^	6.93 ± 0.04 ^Cb^	0.73 ± 0.02 ^Ab^	0.69 ± 0.03 ^ABb^	0.67 ± 0.04 ^Bb^	2.06 ± 0.09 ^Ab^	2.46 ± 0.31 ^Bb^	2.46 ± 0.27 ^Bb^
	DSHP	6.95 ± 0.04 ^Ac^	6.91 ± 0.01 ^ABc^	6.89 ± 0.02 ^Bb^	0.73 ± 0.03 ^Ab^	0.64 ± 0.07 ^Bb^	0.62 ± 0.07 ^Bb^	1.93 ± 0.28 ^Aab^	2.09 ± 0.31 ^Ab^	2.04 ± 0.17 ^Aab^
25:75	None	6.86 ± 0.03 ^Aa^	6.82 ± 0.01 ^Ba^	6.80 ± 0.01 ^Ba^	0.96 ± 0.09 ^Aa^	0.92 ± 0.05 ^Aa^	0.90 ± 0.06 ^Aa^	1.61 ± 0.28 ^Aa^	1.67 ± 0.15 ^Aa^	1.75 ± 0.15 ^Aa^
	TSC	7.04 ± 0.04 ^Ab^	6.99 ± 0.04 ^Bb^	6.93 ± 0.05 ^Cb^	0.70 ± 0.04 ^Ab^	0.66 ± 0.02 ^ABb^	0.64 ± 0.03 ^Bb^	2.00 ± 0.10 ^Aa^	2.45 ± 0.16 ^Bb^	2.65 ± 0.24 ^Bb^
	DSHP	6.97 ± 0.05 ^Ac^	6.94 ± 0.05 ^Ac^	6.88 ± 0.04 ^Bc^	0.71 ± 0.04 ^Ab^	0.60 ± 0.06 ^Bc^	0.61 ± 0.05 ^Bb^	2.02 ± 0.16 ^Aa^	1.89 ± 0.16 ^Aa^	2.49 ± 0.30 ^Bb^
50:50	None	6.89 ± 0.03 ^Aa^	6.84 ± 0.02 ^Ba^	6.81 ± 0.01 ^Ba^	0.92 ± 0.06 ^Aa^	0.90 ± 0.04 ^Aa^	0.87 ± 0.05 ^Aa^	1.80 ± 0.15 ^Aa^	1.88 ± 0.04 ^Aa^	1.88 ± 0.11 ^Aa^
	TSC	7.04 ± 0.03 ^Ab^	7.00 ± 0.02 ^Ab^	6.95 ± 0.04 ^Bb^	0.64 ± 0.05 ^Ab^	0.64 ± 0.04 ^Ab^	0.64 ± 0.03 ^Ab^	2.31 ± 0.19 ^Ab^	2.56 ± 0.12 ^Ab^	2.54 ± 0.40 ^Ab^
	DSHP	6.98 ± 0.05 ^Ac^	6.95 ± 0.04 ^Ac^	6.87 ± 0.04 ^Bc^	0.69 ± 0.02 ^Ab^	0.62 ± 0.02 ^Bb^	0.59 ± 0.04 ^Bb^	2.07 ± 0.09 ^Aab^	2.51 ± 0.35 ^Bb^	2.55 ± 0.64 ^Bb^
75:25	None	6.91 ± 0.04 ^Aa^	6.88 ± 0.01 ^ABa^	6.84 ± 0.00 ^Ba^	0.89 ± 0.06 ^Aa^	0.86 ± 0.06 ^Aa^	0.84 ± 0.07 ^Aa^	1.98 ± 0.04 ^Aa^	2.11 ± 0.09 ^ABa^	2.33 ± 0.10 ^Ba^
	TSC	7.06 ± 0.05 ^Ab^	7.03 ± 0.03 ^Ab^	6.97 ± 0.05 ^Bb^	0.64 ± 0.04 ^Ab^	0.63 ± 0.03 ^Ab^	0.63 ± 0.00 ^Ab^	2.48 ± 0.32 ^Ab^	2.28 ± 0.26 ^Aa^	2.56 ± 0.57 ^Ab^
	DSHP	6.98 ± 0.05 ^Ac^	6.94 ± 0.07 ^Ac^	6.88 ± 0.07 ^Ba^	0.65 ± 0.03 ^Ab^	0.57 ± 0.04 ^Bc^	0.55 ± 0.06 ^Bc^	2.01 ± 0.17 ^Aa^	2.42 ± 0.41 ^Ba^	2.59 ± 0.45 ^Bb^
100:0	None	6.93 ± 0.03 ^Aa^	6.90 ± 0.02 ^Aa^	6.84 ± 0.01 ^Ba^	0.84 ± 0.06 ^Aa^	0.85 ± 0.07 ^Aa^	0.82 ± 0.06 ^Aa^	2.24 ± 0.09 ^Aa^	2.52 ± 0.04 ^ABa^	2.61 ± 0.01 ^Ba^
	TSC	7.08 ± 0.05 ^Ab^	7.04 ± 0.04 ^Ab^	6.99 ± 0.02 ^Bb^	0.63 ± 0.00 ^Ab^	0.59 ± 0.02 ^Ab^	0.57 ± 0.04 ^Bb^	2.77 ± 0.09 ^Ab^	2.62 ± 0.70 ^Aa^	3.00 ± 0.77 ^Ab^
	DSHP	7.00 ± 0.05 ^Ac^	6.96 ± 0.06 ^Ac^	6.88 ± 0.08 ^Bc^	0.63 ± 0.05 ^Ab^	0.58 ± 0.01 ^ABb^	0.50 ± 0.01 ^Bc^	2.01 ± 0.16 ^Aa^	2.34 ± 0.54 ^ABa^	2.57 ± 0.57 ^Ba^

Values are means ± standard deviation. Within the same physicochemical property, means of samples in a row with different superscripts (A, B) differ significantly. Different superscripts (a, b, c) in the same column within the same type of milk indicate a significant difference (*p* < 0.05). Milk blend refers to the ratio of buffalo skim to bovine skim milk.

## Data Availability

The data used to support the findings of this study can be made available by the corresponding author upon request.
